# Ribosome-Inactivating Proteins of *Bougainvillea glabra* Uncovered Polymorphism and Active Site Divergence

**DOI:** 10.3390/toxins13050331

**Published:** 2021-05-04

**Authors:** Yihua Lin, Liting Xu, Yanyan Li, Xiaobin Wu, Yijun Liu, Hongmei Zhu, Hantao Zhou

**Affiliations:** 1 State Key Laboratory of Marine Environmental Science, Xiamen University, Xiamen 361000, China; linyihua@stu.xmu.edu.cn (Y.L.); xuliting@xmu.edu.cn (L.X.); yanyanli2016@stu.xmu.edu.cn (Y.L.); wuxiaobin@stu.xmu.edu.cn (X.W.); 22320181152128@stu.xmu.edu.cn (Y.L.); 2 College of Ocean and Earth Sciences, Xiamen University, Xiamen 361000, China; flyzhu324@163.com

**Keywords:** RIPs, isoforms, polymorphism, active sites, divergence

## Abstract

Ribosome-inactivating proteins (RIPs) are toxic proteins that can inhibit protein synthesis. RIPs purified from *Bougainvillea* have low nonspecific toxicity, showing promise for processing applications in the agricultural and medical fields. However, systematic research on the polymorphism of *Bougainvillea* RIPs is lacking, and it is worth exploring whether different isoforms differ in their active characteristics. The transcriptional and translational expression of type I RIPs in *Bougainvillea glabra* leaves was investigated in this study. Seven RIPs exhibited seasonal variation at both the mRNA and protein levels. The isoforms BI4 and BI6 showed the highest transcriptional expression in both the summer and autumn samples. Interestingly, BI6 was not detected in the protein level in any of the samples. However, the bioinformatics analysis showed that RIPs derived from the same species were gathered in a different cluster, and that the active sites changed among the isoforms during evolution. The significant discrepancy in *Bougainvillea* RIPs mainly locates at both termini of the amino acid sequence, particularly at the C terminus. Post-translational modifications may also exist in *Bougainvillea* RIPs. It is concluded that the reason for the polymorphism of *Bougainvillea* RIPs may be that these proteins are encoded by multiple genes due to genetic processes such as gene duplication and mutation. According to the results of sequence analysis, the possible functional differences of *B. glabra* RIP isoforms are discussed with regard to the observed discrepancy in both active sites and structures.

## 1. Introduction

Ribosome-inactivating proteins (RIPs) are widely expressed in eukaryotes, including plants, fungi, and algae [1,2], and they can be divided into three types according to differences in structure [3]. Type 1 RIPs are single chained, ranging in molecular weight from 25 to 35 kDa. Type 2 RIPs are heterodimeric proteins that contain a type 1 RIP (A-chain) and a lectin domain (B-chain), while type 3 RIPs consist of an A-chain at the N-terminal end and an unknown functional C-terminal domain [4,5]. All RIPs enzymatically release adenine from a single nucleotide in a precise position in a universally conserved GAGA tetraloop of the major rRNA, leading to ribosome inactivation, thus preventing the translation process of protein synthesis and promoting cell apoptosis [2,6]. Plant RIPs can interact with exogenous bacteria or viruses, inactivating the attacker to be inactivated [7,8]. The heterologously expressed RIPs in tobacco plants result in enhanced resistance to *Spodoptera exigua* with low toxicity to the plant cells [9]. Based on these research facts, RIPs have been shown to confer broad-spectrum resistance to a variety of plant diseases and insect pests, especially in the agricultural field [9,10,11]. Furthermore, a formulation of RIPs combined with antibodies was used to treat cancer, indicating that RIPs have anticancer activity and potential promising applications in the cancer medicine field [5,12,13,14].

RIPs have been proved to be polymorphic in many plants [15]. Two saporin types were isolated from *Saponaria officinalis* L. with different toxicities, molecular masses, and amino acid compositions. These isoforms are differentially expressed during leaf development, wounding, and abscisic acid treatment, which suggests that RIP isoforms may play diversified roles during stress responses [16]. Some studies have shown that plants express various RIP isoforms to improve their defense systems to cope with the stress caused by environmental changes [16,17].

The *Bougainvillea* genus is an important landscape and medicinal plant in temperate and subtropical regions, belonging to the family Nyctaginaceae [18,19]. Its genetic information has been analyzed via *Bougainvillea spectabilis* transcriptome sequencing [20]. 

A substance extracted and purified from *Bougainvillea* was identified as a type I RIP by an enzyme activity test [21,22]. The alkaline protein was named bouganin, with a molecular weight of 26.2 kDa. Previous studies indicated that bouganin possesses rRNA N-glycosylase, polynucleotide: adenosine glycosidase (PAG), and antiviral activity, repressing protein synthesis [22,23]. Two RIPs isolated from *Bougainvillea spectabilis* significantly inhibited tobacco mosaic virus (TMV); these RIPs are named Q8 isoform in this study [24]. Meanwhile, *Bougainvillea* RIPs showed a higher activity ratio than other type 1 RIPs, lower toxicity with IC_50_ value 10^−10^ M in a cell-free system, and lower activity on whole cells, implying great application potential [25]. However, systematic research on polymorphisms of *Bougainvillea* RIPs is lacking, and it is worth exploring whether disparate isoforms are different in their active characteristics to find more low-toxicity isoforms. In this study, seven RIP genes were cloned from the leaves of *Bougainvillea glabra*. The expression polymorphism of *Bougainvillea glabra* RIP isoforms at the RNA and protein levels was also examined, and we further predicted the different active sites of RIP isoforms, with the aim of finding more promising isoforms for solving the current problems in medicinal applications and providing a theoretical basis for the further development of *Bougainvillea glabra* RIPs in the field of plant defense. 

## 2. Results

### 2.1. Bougainvillea glabra RIPs Have 3′-End Polymorphism in the Nucleotide Sequence

The mRNA sequences of a precursor RIP with a presumed signal peptide at the N-terminus and a mature RIP from *Bougainvillea glabra*, named BouF and BouM respectively, were cloned by first- and second-phase PCR. The acquisition of seven sequences depended on the 3′-end polymorphism in the nucleotide sequence, while the 5′-end was highly conserved (Figure S1). After sequencing and matching, the seven sequences of the full-length bouganin gene were submitted to the National Center of Biotechnology Information (named BI1 to BI7, with GenBank accession numbers MT478056 to MT478062, respectively). These data show that the RIPs cloned from *Bougainvillea glabra* exhibited polymorphism in the nucleotide sequence, especially at the 3′ end. 

### 2.2. Bougainvillea glabra RIPs Possess Transcriptional Polymorphism 

**Figure 1:**
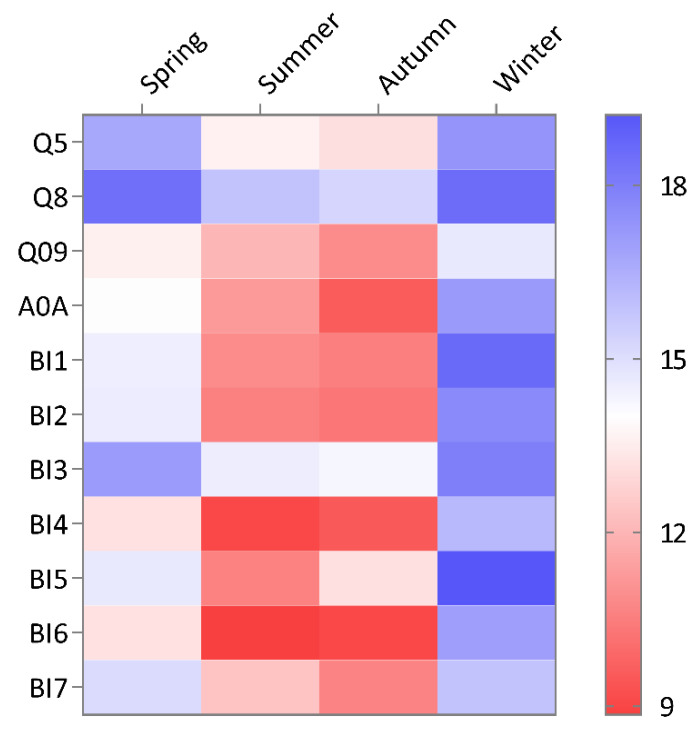
Relative expression of RIPs isoforms of *B. glabra* (BI1-7) and *B. spectabilis* (Q5, Q8, Q09, and A0A) in the four seasons, based on RT-qPCR. A lower threshold value means higher gene expression. The threshold on the right indicates the ΔCt value. The Q5, Q8, Q09, and A0A sequences were downloaded from NCBI.

Comparing RIPs expression across the four seasons, it was found that the expression of all the isoforms was low in spring and winter and high in summer and autumn. We took the ΔCt value, compared with that for a reference gene, as the relative expression of the gene, with a lower threshold meaning a higher expression at the RNA level [26]. The isoforms with the highest expression in summer were BI4 and BI6. The expression of the remaining isoforms peaked in autumn, except for that of BI5 (Figure 1). The Q5, Q8, Q09, and A0A isoforms were isolated from *B. spectabilis* in a previous study [24].

However, it is noteworthy that the relative expression of Q09 was far higher than that of the other isoforms in the winter sample. Q8 exhibited the lowest expression among all the isoforms in spring, summer, and autumn, but the expression of Q8 in winter was slightly higher than that of BI5, which was similar to the expression of BI1 (Figure 1).

### 2.3. Bougainvillea RIPs Have Protein Polymorphism

**Figure 2:**
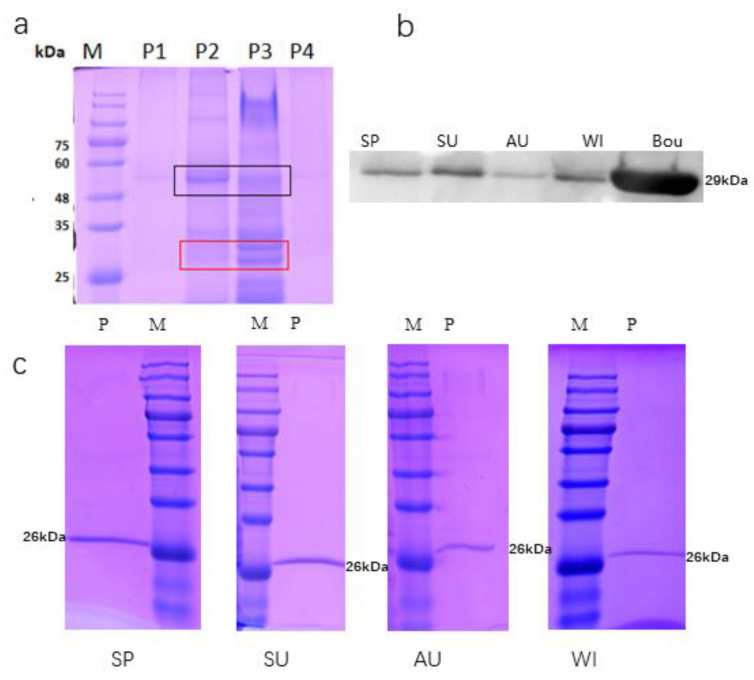
Ribosome-inactivating proteins purified from *Bougainvillea glabra* leaves. (**a**) SDS-PAGE analysis of precipitated proteins. Red squares indicate bouganin strips, and black squares indicate RuBisCo strips. (**b**) Western blot detection of bouganin, where A, B, C, and D indicate spring, summer, autumn, and winter, respectively. Bou represents the recombinant bouganin, approximately 29 kDa. (**c**) Immunoaffinity chromatography of leaf samples in different seasons. M indicates the protein marker, P indicates nature *Bougainvillea* RIPs, and SP, SU, AU, and WI indicate spring, summer, autumn, and winter, respectively.

The affinity constant test indicated that this batch of antibodies had good binding force and could be used for the next step (Table S2). To remove interference by untargeted proteins, we combined ammonium sulfate fractional precipitation with immune affinity chromatography in this study. In the *bougainvillea* blade, the RuBisCo large subunit had the largest precipitation capacity in 25–50% saturated ammonium sulfate. However, the natural *Bougainvillea* RIP belt was mainly concentrated in 50–75% in the corresponding lane (Figure 2b, P3). Hence, the proteins precipitated from 50–75% saturated ammonium sulfate were suitable for immune affinity chromatography in the next step. 

The samples collected from the four seasons were purified by immune affinity chromatography after ammonium sulfate fractional precipitation. A single band was obtained through SDS-PAGE, located in 26 kDa (Figure 2c). The purity of the protein was over 90%. Western blotting also verified that the four single bands were the *Bougainvillea* RIPs (Figure 2b). 

Seven amino acid sequences of *Bougainvillea* RIPs were deduced from the corresponding nucleotide sequences in this study, and four sequences (Q8, Q5, Q09, and A0A) downloaded from NCBI were used as the mass spectrometry database. The results show that the highest and lowest numbers of isoforms were expressed in spring and autumn, respectively. Among these isoforms, BI4, BI5, BI7, A0A, and Q5 were expressed in all four seasons, Q8 and Q09 were expressed in all seasons except autumn, BI1 only appeared in spring and summer, and BI3 only emerged in spring (Table 1), indicating that *Bougainvillea* RIPs have polymorphism at the protein level and are expressed temporally. Interestingly, BI2 and BI6 were not detected in any of the seasons, while BI6 had the highest expression at the RNA level. We found that BI2 and BI6 have shorter amino acid sequences than other isoforms, which may be a reason for the lack of protein expression.

### 2.4. Phylogenetic Analysis and Alignment of Bougainvillea RIPs

**Figure 3:**
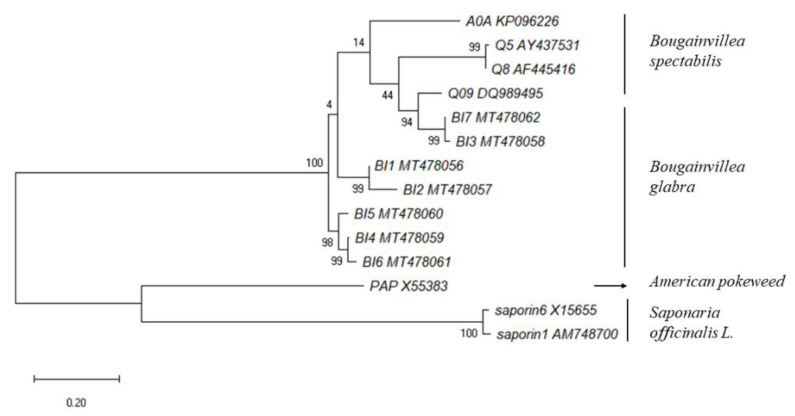
Phylogenetic analysis of *Bougainvillea* RIPs with other typical RIPs based on the amino acid sequences. The phylogenetic tree was constructed with the MEGA 7.0 software, using the maximum likelihood method with 1000 bootstrap replicates.

We constructed a phylogenetic tree to analyze the genetic relationship between *Bougainvillea* RIPs and other typical RIPs (GenBank No. in Table 2). The tree indicated that BI4, BI5, and BI6 were gathered in a cluster, while other isoforms of *Bougainvillea* RIPs formed the same branch. BI3 and BI7 had a closer genetic relationship with Q09, which was located in a different cluster with A0A. Moreover, BI1 to BI7 were the isoforms derived from *Bougainvillea glabra*, while other isoforms were isolated from *Bougainvillea spectabilis* (Figure 3). Interestingly, the isoforms isolated and purified from the same phenotype were located on different branches, indicating that these homologous genes were amplified specifically in different species after the differentiation of *Bougainvillea*.

**Figure 4:**
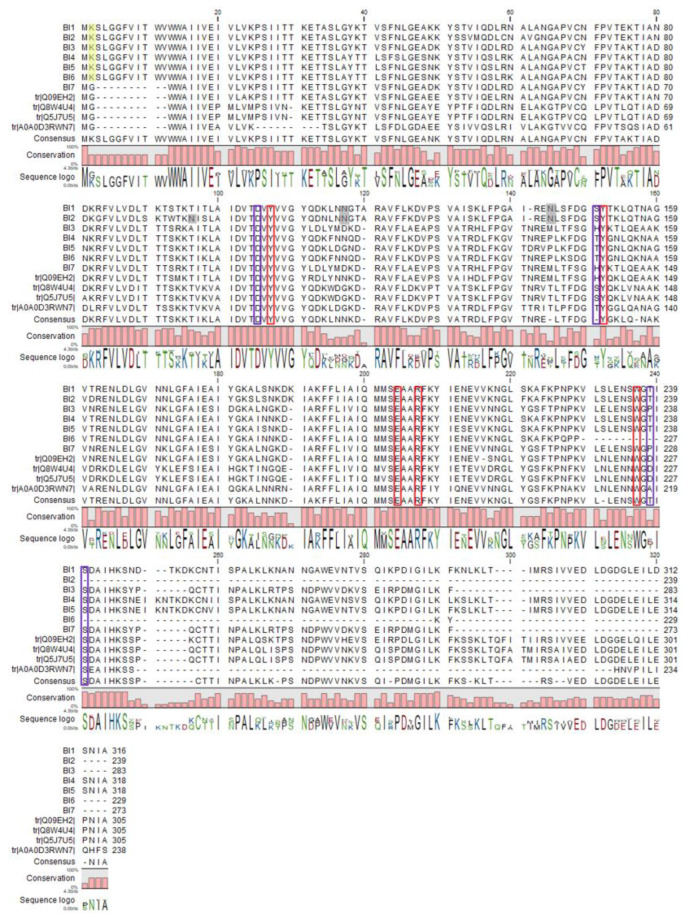
Sequence alignment of *Bougainvillea* RIPs was performed via the CLC main workbench combined with the Sequence logo. The red frames indicate N-glycosidase active sites, while the blue frames indicate the sites involved in stabilizing enzyme substrate interaction. The gray background shows the predicted glycosylation site, and the yellow background indicates predicted acetylation sites.

**Figure 5:**
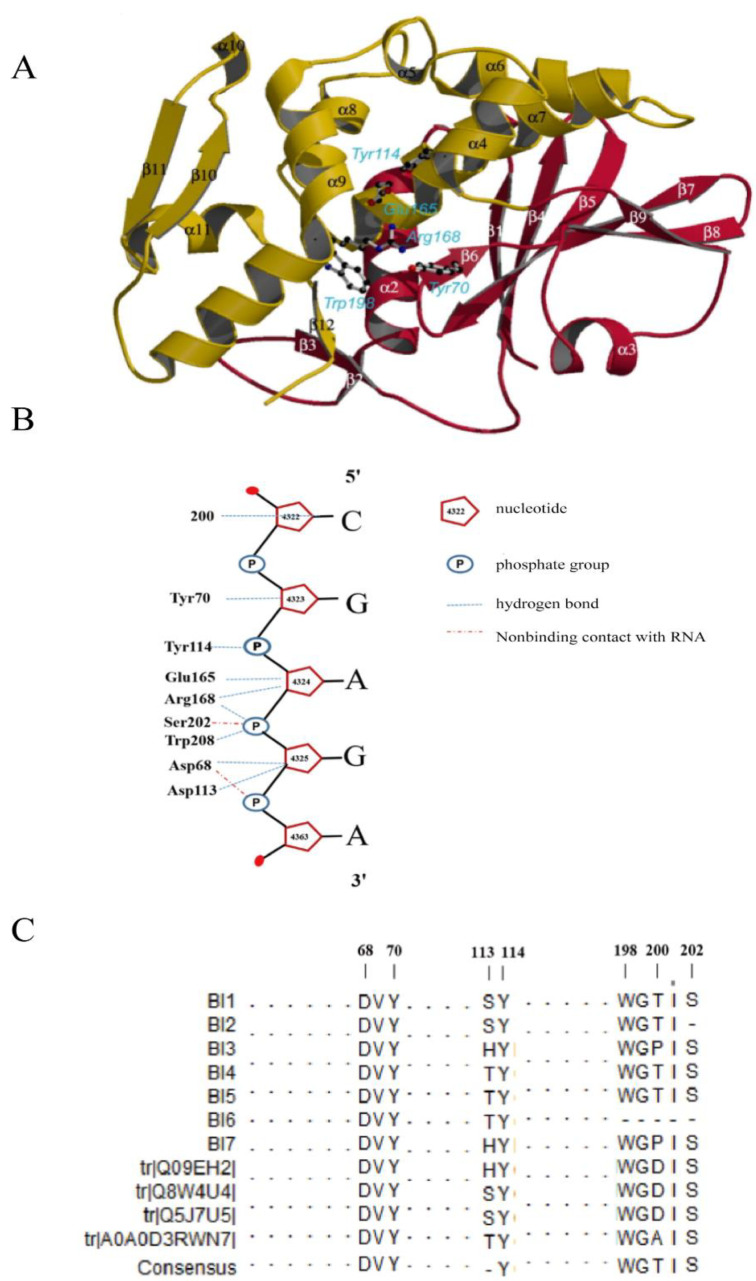
Schematic diagram of Bougainvillea RIPs’ key residue sites. **A** shows the spatial structure of bouganin, which was published in Fermani et al. (2009) [21]. The red and yellow parts indicate the N- and C-terminal regions, respectively. **B** represents the interaction between the amino acids of the active site and rRNA (SRL loop)., while **C** indicates the key active sites of *Bougainvillea* RIPs.

Furthermore, we aligned the amino acid sequences of *Bougainvillea* RIPs, and the Sequence logo was combined to reflect the degree of conservation of each locus (Figure 4). The consistency of the *Bougainvillea* RIPs was 58–99% according to the blast comparison results (Table 2). They showed that the similarity of the central region is higher than that of the two terminal regions, and the central region is the functional structure domain of the RIPs, indicating that *Bougainvillea* RIPs may have similar functions [21] (Figure 5A). 

A large number of studies have found that the RIP family exhibits five highly conserved amino acid residues, referring to the activity of N-glucosidase, which are the key to the enzymatic activity of RIP [27,28,29]. The five key loci are Tyr70, Tyr114, Glu165, Arg168, and Trp198 in Q8. Figure 4 shows that these five active sites of *Bougainvillea* RIPs are highly conserved, except that BI6 lacks Trp198. It has been reported that effective interaction between the enzyme and the substrate determines the deadenylation of different adenine substrates by ribosomal inactivated proteins and the inhibition of protein synthesis (Figure 5B), while the interaction between the enzyme and the substrate mainly depends on the conserved N-glucosidase active site and the auxiliary site [21]. Some conserved amino acid residues around the RIP active region also play an important role, participating in the stabilization of the enzyme substrate interaction. These residues correspond to Asp68, Asp113, Lys200, and Ser202 in the Q8 isoform (bouganin) (Figure 5C). The results show that Asp113 and Lys200 were not conserved among the *Bougainvillea* RIPs. For the Asp113 lous, the isoforms BI1, BI2, BI4, BI5, BI6, and A0A showed a change from acidic Asp to alkaline Lys, and BI3, BI7, and Q09 showed a change to non-polar hydrophobic Ala. However, for Lys200, the Q5, Q8, and Q09 isoforms showed replacement by Asp; A0A, by Ala; BI3 and BI7, by Pro; and the rest, by Thr (Figure 4). It is noteworthy that the BI2 and BI6 sequences terminate translation in advance, so they lack these auxiliary active sites. 

### 2.5. RIP Post-Translational Modification Predictions 

The glycosylation site prediction for the *Bougainvillea* RIPs showed that only BI1 and BI2 may exhibit glycosylation modification with a potential value greater than the threshold; BI1 has two possible sites (117 and 144 positions), and BI2 has three loci (96, 117, and 144 positions) (Table 3 and Figure 4 with gray background). All the *Bougainvillea* RIPs may possess serine, threonine, and tyrosine phosphorylation on three types of sites, but each isoform has a different modification locus position and number. Among the isoforms, Q8 and Q5 have the highest numbers of phosphorylation sites. However, A0A has the fewest phosphorylation modification loci (Table 4). Only BI1 to BI6 might have acetylation modification sites (Table 4 and Figure 4 with yellow background). The three-dimensional structures of the *Bougainvillea* RIPs demonstrated that the A0A, BI2, and BI6 isoforms vary wildly from the other isoforms, which is mainly reflected in the C terminus ending in an α spiral, lacking the folding clip (Figure S1). 

## 3. Discussion

The *Bougainvillea* RIPs possess transcriptional polymorphism, and all the isoforms exhibit diversified expression in the different seasons. It was previously shown that the expression of each isoform is affected by a cis-acting element that combines with the transcription factor under jasmonic acid stress [17]. Besides this, the *Bougainvillea* RIPs have a close genetic relationship with PAP, which alters the splicing of viral RNA and reduces viral infection [30,31]. Hence, we speculated that the expression of each isoform might be caused by the different transcriptional regulatory mechanisms of RIP isoforms. Additionally, isoform expression was regulated by the season; that is, the RNA expression levels of *Bougainvillea* RIPs are seasonally specific. Furthermore, the expression of the majority of the isoforms was higher in summer and autumn than in spring and winter. This phenomenon was also found for PAP [15] and is usually related to environmental factors, such as pests, drought, and high temperature. Rippmann et al. also reported that RIP transcripts were induced upon environmental stress [32]. Frequent periods of plant pests, drought, and high temperature are present in the summer and autumn; in this situation, RIPs play a pivotal role in the plant’s defense as an antiviral and pest-resisting toxic protein under stress.

The RIPs’ diverse seasonal expression was also detected at the protein level, which was consistent with that at the transcript level. Parente et al. (2008) proposed that seasonal regulation is the main reason for the differential seasonal expression of RIPs [15]. An increase in temperature promotes the activity of enzymes in plants [33], and summer is a period marked by pests and high temperatures, while in autumn, the drought stress on the plants intensifies. As a result, the maximum expression at the transcriptional and translational levels of *Bougainvillea* RIPs appeared in summer or autumn. 

It is worth noting that the high transcriptional expression of isoforms in summer and autumn did not translate to the high expression of the corresponding proteins, indicating that not all the isoforms could eventually be translated into proteins, which may be related to differences in their ability to resist seasonal changes—that is, the differences in their active functions. Some unknown and complex mechanisms may lead to the transcriptional difference and protein non-expression, which may explain why BI2 and BI6 were not translated in the four seasonal samples. Furthermore, the specific mRNA splicing events significantly impact the protein abundance levels [34], which may be explain why the isoforms expressed in mRNA and did not translate in protein. 

To determine whether these isoforms performed different biological functions, we further used bioinformatics to explore the evolution and active sites among *Bougainvillea* RIPs. The central region of each isoform was highly conserved, and the five conserved N-glucosidase active sites of the RIPs were retained. Nevertheless, some sites at both termini of the sequence were less conserved; this may be the result of multiple genetic processes such as fusion and deletion of mutated genes during the evolution of RIP [35,36,37]. RIP first appeared before the common ancestor of eukaryotes and bacteria and fused with the lectin of bacteria in the process of evolution. There are seven collateral homologues in *Oryza sativa* RIPs, indicating that at least six gene duplication and differentiation events occurred in RIPs. Therefore, in the process of overall evolution, RIP may have undergone repeated differentiation and other genetic processes several times. Besides this, RIPs are proteins encoded by a polygenic family [38], which is an important cause of gene polymorphism. 

Effective interaction between the enzyme and substrate is necessary for the deadenylation of different adenine substrates by RIPs and the inhibition of protein synthesis [21]. However, the interaction between the enzyme and the substrate mainly depends on the conserved N-glucosidase active site and the auxiliary site. BI6 was found to be missing the active residues of tryptophan (i.e., Q8’s Trp198), while the other isoforms retained the five highly conserved active amino acid residues of the RIP family in *Bougainvillea*. The common characteristic of these auxiliary sites is the ability to stabilize the active site catalysis of adenine and provide good conditions for reaction with substrates by interacting with the phosphate groups of the RNA backbone [39,40,41,42]. After the protonation of adenine, the conserved active amino acid Tyr70 side chain was rotated, and adenine was embedded in the active region, facilitating the next adenosine removal reaction. In *Bougainvillea* RIPs, the corresponding point changed to different degrees in all except Q5. Changes in the physicochemical properties of amino acids may affect the interactions between atoms. The BI2, BI6, and A0A isoforms ended with an alpha spiral at the C terminus and lacked the fold segment in the 3D modeling. However, the 3D structure of the other isoforms were consistent with the Q8 isoform, ending in a fold (Figure S3). Three RIPs were isolated from the bulbs of *Muscari armeniacum* L. and Miller and named MU1-3. Only MU1 and MU2 contain glycation sites, and both inhibit protein synthesis more strongly than MU3 (the IC_50_ values were 7 ng/mL, 9.5 ng/mL, and 4 ng/mL, respectively) [43]. A similar phenomenon was found in the study of pokeweed antiviral protein (PAP) [44]. Therefore, the effect of the deoxyadenosine may be affected by the non-conservation of *Bougainvillea* RIPs at this site. Combined with the sequence alignment results in this paper, we hypothesized that the differences in the key sites of *Bougainvillea* RIPs would lead to differences in their biological functions. 

## 4. Materials and Methods

### 4.1. Plant Material 

The samples of *Bougainvillea glabra* were collected at Xiamen University in July 2018 (summer), November 2018 (autumn), January 2019 (winter), and April 2019 (spring).

### 4.2. RNA Extraction and PCR Amplification

Total RNA was extracted from 100 milligrams of leaf samples using an RNAprep Pure Plant Kit (Tiangen Biotech, China). The first-strand cDNA was synthesized with Oligo primer by using a Revert Aid First Strand cDNA Synthesis Kit (Thermo Fisher Scientific, USA) following the manufacturer’s instructions, using the oligo dT primer for the 3′ RACE and the SP1 primer for the 5′ RACE. Two-stage PCR was used for bouganin gene amplification; the cDNA was used in the first stage, and the PCR product was used as the template in the second phase. Additionally, 1×PCR buffer, 5.0 nmol dNTP, 2 μmol forward and reverse primers, and 1 U Taq polymerase were added in the PCR reaction system. Then the PCR programs were conducted with an initial denaturation at 94 °C for 2 min; 30 cycles of 94 °C for 30 s, 60 °C for 30 s, and 72 °C for 1 min; and a final 72 °C extension for 10 min for both PCR rounds. The 3′ RACE and 5′ RACE (5′/3′RACE Kit, 2nd generation) were conducted according to the manufacturer’s user guide. The primer sequences are listed in Table S1. Finally, the 5-GSP-CO and 3-GSP series primers were used to acquire full-length bouganin mRNA sequences. 

### 4.3. RT-qPCR

The specific qPCR primers were designed using the PRIMER5.0 software and synthesized by Sangon Biotech Co., Ltd., Shanghai, China (shown in Table S1). The Q8W4U4, Q5J7U5, A0A0D3RWN7, and Q09EH2 sequences were downloaded from NCBI and abbreviated as Q8, Q5, A0A, and Q09, respectively. *Bougainvillea glabra* 18s rRNA served as the internal control.

PCR was performed in a final volume of 20 µL, which contained 50 ng of cDNA, 10 µL of 2×TB Green Premix Ex Taq (Tli RNaseH Plus), 0.4 µL of ROX Reference dye II, and 2 pmol each of the forward and reverse primers (Takara, Dalian, China). The PCR programs were conducted at 95 °C for 20 s, followed by 40 cycles of 95 °C for 5 s and 60 °C for 30 s. Ct values were calculated based on technical triplicate experiments performed on three biological replicates. The mRNA levels were quantified using the ∆Ct method. A melting curve analysis was performed to confirm the presence of a single PCR product after each reaction.

### 4.4. Anti-Bouganin Antibody Preparation

The gene encoding bouganin (the Q8 isoform) was cloned into a pET-28a plasmid using the ClonExpress II One Step Cloning Kit (Vazyme, China). The recombinant pET-28a-Bou plasmid was transferred to *E. coli* BL21 and induced to express the bouganin protein under 1mM IPTG. The recombinant bouganin protein was sent to the Experimental Animal Center of Xiamen University for the preparation of a rabbit antibody, for which 4 mg of purified bouganin protein was used in one rabbit with the injection of 1 mg per immunization, once a week, 4 times to obtain the antiserum.

The appropriate amount of CNBr-activated Sepharose 4B powder was dissolved in 1 mM HCl and loaded into the column immediately. The rabbit anti-bouganin antibody was purified with an immune affinity column, and then, the high-purity anti-bouganin was used for coupling with CNBr-activated Sepharose 4B to prepare the immune affinity chromatography column. Anti-bouganin antibody was detected by ELISA. After the column preservation solution was drained out, it was cleaned with 5 times the column volume of ultrapure water and balanced with 10 times the column volume of phosphate buffer saline (PBS). 

### 4.5. Protein Isolation from Bougainvillea glabra 

A quantity of 10 g of *Bougainvillea glabra* leaves was ground in liquid nitrogen and homogenized overnight in 50 mL of extraction buffer, including 150 mM 4×Tris-HCl (pH = 8.0), 10% glycerin, 100 mM NaCl, 5 mM EDTA, and 0.5% Tween-20. Then the sample was centrifuged for 20 min at 16,000× *g* at 4 °C (R20A2 rotor, Hitachi, Tokyo, Japan). The supernatant was used for SDS-PAGE and further study. 

### 4.6. Ammonium Sulfate Fraction

After simple filtration through cheesecloth, the supernatant was fractionated with ammonium sulfate stirred 30 min and centrifuged for 10 min at 10,000 g. The supernatant was precipitated with different saturability ranges (0–25%, 25–50%, 50–75%, and 75–100%). The pellets were suspended in 5 mL of PBS and named P1, P2, P3, and P4, respectively. The residual insoluble substance was removed by another centrifugation again and desalinated by ultrafiltration. All the extraction procedures were conducted at 4 °C.

### 4.7. Immunoaffinity Chromatography 

The P3 fraction (50–75%) was loaded into the anti-bouganin immune affinity column. The column was closed and incubated overnight at 4 °C to ensure that the protein was fully absorbed on the affinity column. Then, the sample was collected and washed with at least 10 times the volume of PBS. The target protein was eluted with Gly-HCl (pH 2.74) and the permeable fluid was collected and neutralized with Tris-HCl (1M). The samples were collected during the immune affinity chromatography washing and elution phase and detected by SDS-PAGE.

### 4.8. Protein Polymorphism Analysis and Western Blotting

SDS-PAGE was performed in a 10% separating gel according to the manual (Bio-Rad, USA). The protein bands were visualized on the gel by Coomassie Brilliant Blue staining (Solarbio). The amount of protein at all steps of purification was estimated according to the method of Bradford using a Solarbio protein assay kit. The target protein bands were cut, washed in double distilled water, and identified using a timsTOF-Pro. The timsTOF-Pro (Bruker, Bremen, Germany) was operated in PASEF mode using Compass Hystar 5.0.36.0. The parameters were as follows: mass range, 100 to 1700 m/z; 1/K0 start, 0.6 V⋅s/cm^2^; end, 1.6 V⋅s/cm^2^; ramp time, 110.1 ms; lock duty cycle to 100%; capillary voltage, 1600 V; dry gas, 3 L/min; dry temperature, 180 °C; PASEF settings: 10 MS/MS scans (total cycle time, 1.27 s); charge range, 0–5; active exclusion for 0.4 min; scheduling target intensity, 10,000; intensity threshold, 2500; and CID collision energy, 42 eV. 

All the raw files were analyzed by PEAKS Studio X software (Bioinformatics Solutions Inc., Waterloo, ON, Canada). The data were searched against the mass spectrometry database (BI1-BI7, Q8, Q5, Q09, and A0A, a total of 11 sequences). De novo sequencing of peptides, database searching, and characterization-specific PTMs were used to analyze the raw data; the false discovery rate (FDR) was set to ≤1%, and [−10 * log(*p*)] was calculated accordingly, where *p* is the probability that an observed match is a random event. The PEAKS used the following parameters: (i) precursor ion mass tolerance, 10 ppm; (ii) fragment ion mass tolerance, 0.02 Da (the error tolerance); (iii) tryptic enzyme specificity with two missed cleavages allowed; (iv) monoisotopic precursor mass and fragment ion mass; (v) a fixed modification of cysteine carbamidomethylation; and (vi) variable modifications, including the N-acetylation of proteins and oxidation of Met.

Western blot analysis was conducted to examine the Bouganin expression in tissues using anti-bou antibody (1:1000). An aliquot of protein from each sample was resolved by 10% SDS-PAGE and electrotransferred to a PVDF membrane for standard Western blot analysis. 

### 4.9. Bioinformatics Analysis

The amino acid sequences of individual isoforms published in NCBI GenBank (Table 2) were examined separately with DNAMAN v6 and aligned using ClustalX 2.0. The phylogenetic tree was constructed with MEGA 7.0 software, using the maximum likelihood method with 1000 bootstrap replicates. Multiple sequence alignment was performed using the CLC main workbench combine with the Sequence logo. The NetNElvc 1.0 server, Netphos 2.0 server, and NetAcet 1.0 server were used to predict the possible post-modification sites of the RIPs. In the SWISS-MODEL, a homologous modeling method was used to predict the three-dimensional structures of the *Bougainvillea* RIPs.

## Figures and Tables

**Table 1:** Mass spectrometry results for *Bougainvillea* RIPs.

Isoforms	Spring	Summer	Autumn	Winter	Isoforms	Spring	Summer	Autumn	Winter
BI1	+	+	-	-	BI7	+	+	+	+
BI2	-	-	-	-	A0A	+	+	+	+
BI3	+	-	-	-	Q5	+	+	+	+
BI4	+	+	+	+	Q8	+	+	-	+
BI5	+	+	+	+	Q09	+	+	-	+
BI6	-	-	-	-					

**Table 2:** Identity of *Bougainvillea* RIPs and other RIPs.

Protein Name	Organism	GenBank No.	BI1	BI2	BI3	BI4	BI5	BI6	BI7	Q8	A0A	Q5	Q09	Saporin1	Saporin6	PAP
BI1	*Bougainvillea glabra*	MT478056	100.0													
BI2	*Bougainvillea glabra*	MT478057	93.3	100.0												
BI3	*Bougainvillea glabra*	MT478058	75.8	73.4	100.0											
BI4	*Bougainvillea glabra*	MT478059	86.0	78.5	75.3	100.0										
BI5	*Bougainvillea glabra*	MT478060	87.3	79.3	74.9	95.0	100.0									
BI6	*Bougainvillea glabra*	MT478061	82.5	75.9	77.3	97.4	92.6	100.0								
BI7	*Bougainvillea glabra*	MT478062	75.3	72.2	98.2	74.7	74.4	76.7	100.0							
Q8	*Bougainvillea spectabilis*	AF445416	61.9	62.4	72.8	61.1	62.8	65.1	74.3	100.0						
A0A	*Bougainvillea spectabilis*	KP096226	67.4	65.1	66.4	70.6	71.4	71.4	68.1	67.2	100.0					
Q5	*Bougainvillea spectabilis*	AY437531	61.5	61.9	72.1	61.1	62.8	65.1	73.5	99.0	66.8	100.0				
Q09	*Bougainvillea spectabilis*	DQ989495	70.6	73.9	87.1	68.1	67.1	75.7	89.0	74.3	68.8	73.7	100.0			
Saporin1	*Saponaria officinalis* L.	AM748700	24.5	28.1	23.8	24.9	24.6	28.4	24.2	23.0	29.5	23.0	23.7	100.0		
Saporin6	*Saponaria officinalis* L.	X15655	25.9	29.0	24.3	26.0	25.6	29.3	24.7	24.8	29.9	24.8	25.6	96.5	100.0	
PAP	*American pokeweed*	X55383	31.1	32.3	33.0	30.3	31.0	31.0	33.3	29.4	31.9	29.4	29.1	26.6	28.1	100.0

**Table 3:** Prediction sites for glycosylation modification of *Bougainvillea* RIPs.

SeqName	Position	Potential	Jury Agreement	N-Glyc Result
BI1	117 NGTA	0.7119	(9/9)	++
BI1	144 NLSF	0.6681	(9/9)	++
BI2	96 NISL	0.5981	(6/9)	+
BI2	117 NGTA	0.5583	(6/9)	+
BI2	144 NLSF	0.6458	(9/9)	++

**Table 4:** Predicted acetylation modification and phosphorylation modification sites of *Bougainvillea* RIPs.

Gene Name	Acetylation Modification Sites			No. of Phosphorylation Modification Sites			
	Context	Score	Acetylation	Serine	Threonine	Tyrosine	Total
BI1	-MKSLGG	0.508	YES	4	4	4	12
BI2	-MKSLGG	0.508	YES	6	1	6	13
BI3	-MKSLGG	0.508	YES	4	5	7	16
BI4	-MKSLGG	0.508	YES	7	5	4	16
BI5	-MKSLGG	0.508	YES	7	4	4	15
BI6	-MKSLGG	0.508	YES	3	4	5	12
BI7	-MGWWA	0.464	NO	4	5	7	16
Q8	-MGWWA	0.464	NO	8	7	4	19
Q09	-MGWWA	0.464	NO	4	4	6	14
Q5	-MGWWA	0.464	NO	8	7	4	19
A0A	-MGWWA	0.464	NO	3	2	3	8

## Data Availability

The sequences of BI1-BI7 were uploaded in GenBank database with No. MT478056 -MT478062.

## Supplementary Materials

The following are available online at https://www.mdpi.com/article/10.3390/toxins13050331/s1: Figure S1: The the 3′ end nucleotide sequence alignment of *B. glabra* isoforms; Figure S2: Antibody coupling effect detection; Figure S3: The three-dimensional structures prediction of the Bougainvillea RIPs. Table S1: Primer sequences used in this study, Table S2: The examination results of ELISA analysis of anti-Bou.
