# Supplementary material for: Ribosome-Inactivating Proteins of Bougainvillea glabra Uncovered Polymorphism and Active Site Divergence
Source: Toxins (Basel). 2021 May 4;13(5):331. doi: 10.3390/toxins13050331 (PMC8147849; doi:10.3390/toxins13050331)
Supplement: Supplementary file 1 [file toxins-13-00331-s001.zip › SUPP FINAL.pdf]

Ribosome-Inactivating Proteins of *Bougainvillea glabra* Uncovered Polymorphism and Active Site Divergence

Yihua Lin, Liting Xu, Yanyan Li, Xiaobin Wu, Yijun Liu, Hongmei Zhu and Hantao Zhou

Table S1. Primer sequences used in this study.

Primer	Sequence (5′—3′)	Function
Q09-F	GAGGCCGAAGAG-TACTCCACTG	RT-qPCR for detecting the RIPs polymor-phism
Q09-R	AA-TAG-TTTTCATCGAGGTCG-	
A0A-F	AGCTTTGACCTTGGA-GACGC	
A0A-R	CGGCTCGATCTTT-GCCATCC	
Q5-F	CCTCACTT-GGTTACAACACTGTGTC	
Q5-R	CCCTTAGCCAATTCATT-GCG	
Q8-F	CACCTCACTT-GGTTACAACACCG	
Q8-R	GTGTGCCCTTAGCCAATTCG	
BI1-F	AGCCAATGACAA-GCGATTTGTTC	
BI1-R	ATTGAGTTTGTCTTGA-TAACCCACC	
BI2-F	CCAATGACAAGGGATTT-GTTCTAG	
BI2-R	GGGTTT-GAATGCTTTAAA-TAATCCG	
BI3-F	GGAAAGCTATTACGCTT-GCTATAGA	
BI3-R	GACGGGTTGCAACAC-TAGGAGC	
BI4-F	ATGTG-TATGTGGTCGGTTATCAAG	
BI4-R	CCATCAAATTTTAAC-GGTTCACG	
BI5-F	AAACCATAGCCGA-TAACAAGCG	
BI5-R	CATTGCCATCCAATTT-GTCTTG	

 https://doi.org/10.3390/toxins13050331

BI6-F	AAATCTGATGGAACTTACGGAAACC	
BI6-R	CAATGTACTTGAATCGTGCTGCC	
BI7-F	CAAGAGGCTGCCAAAGTGAATAGAG	
BI7-R	CAATATACTTGAATCGTGCTGCCTC	
18s rRNA-F	CCATAAACGATGCCGACCAG	
18s rRNA-R	GCCTTGCGACCATACTCCC	
Bou-F-FOR	AATACCATGGGTTGGTGGGCTATCAT	First-stage PCR for bouganin amplification
Bou-F-REV	ATGGGTAACCATTAGGCAATGTTTGGCTC	
Bou-M-FOR	ATACCATCCATGGACAACACCGTGTCAT	Second-stage PCR for bouganin amplification
Bou-M-REV	TTAGGTCACCTTATTTGGAGCTTTTAAACT	
Oligo (dT)-Anchor Primer	GACCACGCGTATCGATGTCGACT(16)V	cDNA synthesis in 3' race, dA-tailed cDNA 1st-amplification
PCR Anchor Primer	GACCACGCGTATCGATGTCGAC	3' race PCR
SP1	CACTTTGGCAGCCTCTTGAAGGGTTT	cDNA synthesis in 5' race
SP2	CCTGGGAAAAGATGACGGGTTGC	dA-tailed cDNA 1st amplification
SP3	CGCTTGTCATCGGCTATGGTTT	dA-tailed cDNA 2nd amplification
SP5	ATGATGTCAGAGGCAGCACGATTC	3' race PCR
bou-3RACE-GSP	AAATGATGTCAGAGGCAGCACGA	3' race primer
bou-5RACE-GSP	CAGAAATCAAAATCATAAACACACATCCT	5' race primer
bou-5RACE-SP3	CGCTTGTCATCGGCTATGGTTTTTG	5' race primer
5-GSP-CO	AAAGCCA TCAA TAA TAACCACC	Full-length Bouganin gene amplification
3-GSP1	TTAACCACCTCA TTCTGAA TA TAAA	
3-GSP2	TGTTACATTTATCTTTTGTATCATTTG	
3-GSP3	ATTTATTTATTTAAGCTAGTGCTGG	

3-GSP4	TTTTTTACAGA TCACAA TA TTACA TTAC
3-GSP5	TTTAAAA-GCAGAAAAGTAATGA-GAT
3-GSP6	TTTTTTGATGAGAT-TATTGCACTAT

Table S2. The examination results of ELISA analysis of anti-Bou.

Serum Dilution Ratio	1k	2k	4k	8k	16k	32k	64k	128k	256k	512k	1000k	negative	blank
A450 nm	1.278	1.155	1.059	0.895	0.706	0.567	0.43	0.294	0.192	0.141	0.116	0.051	0.048
A450 nm/blank	26.63	24.06	22.06	18.65	14.71	11.81	8.95	6.11	4	2.93	2.42		

Note: the value below 2.5 is marked with red; 1k = 1000.

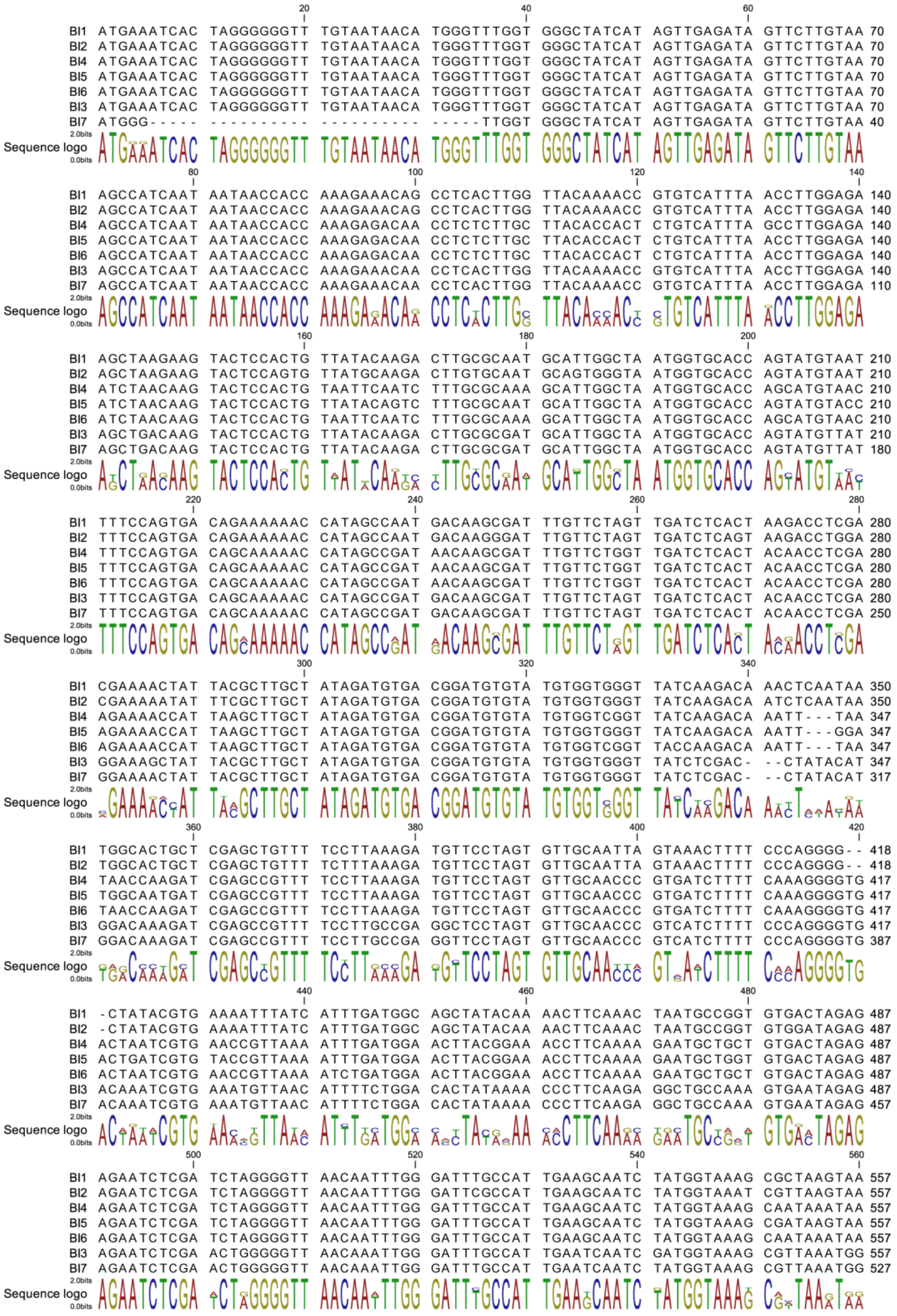
20 40 60
BI1 ATGAAATCAC TAGGGGGGTT TGTAATAACA TGGGTTTGGT GGGCTATCAT AGTTGAGATA GTTCTTGTAA 70
BI2 ATGAAATCAC TAGGGGGGTT TGTAATAACA TGGGTTTGGT GGGCTATCAT AGTTGAGATA GTTCTTGTAA 70
BI4 ATGAAATCAC TAGGGGGGTT TGTAATAACA TGGGTTTGGT GGGCTATCAT AGTTGAGATA GTTCTTGTAA 70
BI5 ATGAAATCAC TAGGGGGGTT TGTAATAACA TGGGTTTGGT GGGCTATCAT AGTTGAGATA GTTCTTGTAA 70
BI6 ATGAAATCAC TAGGGGGGTT TGTAATAACA TGGGTTTGGT GGGCTATCAT AGTTGAGATA GTTCTTGTAA 70
BI3 ATGAAATCAC TAGGGGGGTT TGTAATAACA TGGGTTTGGT GGGCTATCAT AGTTGAGATA GTTCTTGTAA 70
BI7 ATGGG----- ---------- ---------- -----TTGGT GGGCTATCAT AGTTGAGATA GTTCTTGTAA 40
Sequence logo
80 100 120 140
BI1 AGCCATCAAT AATAACCACC AAAGAAACAG CCTCACTTGG TTACAAAACC GTGTCATTTA ACCTTGGAGA 140
BI2 AGCCATCAAT AATAACCACC AAAGAAACAG CCTCACTTGG TTACAAAACC GTGTCATTTA ACCTTGGAGA 140
BI4 AGCCATCAAT AATAACCACC AAAGAGACAA CCTCTCTTGC TTACACCACT CTGTCATTTA GCCTTGGAGA 140
BI5 AGCCATCAAT AATAACCACC AAAGAGACAA CCTCTCTTGC TTACACCACT CTGTCATTTA ACCTTGGAGA 140
BI6 AGCCATCAAT AATAACCACC AAAGAGACAA CCTCTCTTGC TTACACCACT CTGTCATTTA ACCTTGGAGA 140
BI3 AGCCATCAAT AATAACCACC AAAGAAACAA CCTCACTTGG TTACAAAACC GTGTCATTTA ACCTTGGAGA 140
BI7 AGCCATCAAT AATAACCACC AAAGAAACAA CCTCACTTGG TTACAAAACC GTGTCATTTA ACCTTGGAGA 110
Sequence logo
160 180 200
BI1 AGCTAAGAAG TACTCCACTG TTATACAAGA CTTGCGCAAT GCATTGGCTA ATGGTGCACC AGTATGTAAT 210
BI2 AGCTAAGAAG TACTCCAGTG TTATGCAAGA CTTGTGCAAT GCAGTGGGTA ATGGTGCACC AGTATGTAAT 210
BI4 ATCTAACAAG TACTCCACTG TAATTCAATC TTTGCGCAAA GCATTGGCTA ATGGTGCACC AGCATGTAAC 210
BI5 ATCTAACAAG TACTCCACTG TTATACAGTC TTTGCGCAAT GCATTGGCTA ATGGTGCACC AGTATGTACC 210
BI6 ATCTAACAAG TACTCCACTG TAATTCAATC TTTGCGCAAA GCATTGGCTA ATGGTGCACC AGCATGTAAC 210
BI3 AGCTGACAAG TACTCCACTG TTATACAAGA CTTGCGCGAT GCATTGGCTA ATGGTGCACC AGTATGTTAT 210
BI7 AGCTGACAAG TACTCCACTG TTATACAAGA CTTGCGCGAT GCATTGGCTA ATGGTGCACC AGTATGTTAT 180
Sequence logo
220 240 260 280
BI1 TTTCCAGTGA CAGAAAAAAC CATAGCCAAT GACAAGCGAT TTGTTCTAGT TGATCTCACT AAGACCTCGA 280
BI2 TTTCCAGTGA CAGAAAAAAC CATAGCCAAT GACAAGGGAT TTGTTCTAGT TGATCTCAGT AAGACCTGGA 280
BI4 TTTCCAGTGA CAGCAAAAAC CATAGCCGAT AACAAGCGAT TTGTTCTGGT TGATCTCACT ACAACCTCGA 280
BI5 TTTCCAGTGA CAGCAAAAAC CATAGCCGAT AACAAGCGAT TTGTTCTGGT TGATCTCACT ACAACCTCGA 280
BI6 TTTCCAGTGA CAGCAAAAAC CATAGCCGAT AACAAGCGAT TTGTTCTGGT TGATCTCACT ACAACCTCGA 280
BI3 TTTCCAGTGA CAGCAAAAAC CATAGCCGAT GACAAGCGAT TTGTTCTAGT TGATCTCACT ACAACCTCGA 280
BI7 TTTCCAGTGA CAGCAAAAAC CATAGCCGAT GACAAGCGAT TTGTTCTAGT TGATCTCACT ACAACCTCGA 250
Sequence logo
300 320 340
BI1 CGAAAACTAT TACGCTTGCT ATAGATGTGA CGGATGTGTA TGTGGTGGGT TATCAAGACA AACTCAATAA 350
BI2 CGAAAAATAT TTCGCTTGCT ATAGATGTGA CGGATGTGTA TGTGGTGGGT TATCAAGACA ATCTCAATAA 350
BI4 AGAAAACCAT TAAGCTTGCT ATAGATGTGA CGGATGTGTA TGTGGTCGGT TATCAAGACA AATT---TAA 347
BI5 AGAAAACCAT TAAGCTTGCT ATAGATGTGA CGGATGTGTA TGTGGTGGGT TATCAAGACA AATT---GGA 347
BI6 AGAAAACCAT TAAGCTTGCT ATAGATGTGA CGGATGTGTA TGTGGTCGGT TACCAAGACA AATT---TAA 347
BI3 GGAAAGCTAT TACGCTTGCT ATAGATGTGA CGGATGTGTA TGTGGTGGGT TATCTCGAC- --CTATACAT 347
BI7 GGAAAACTAT TACGCTTGCT ATAGATGTGA CGGATGTGTA TGTGGTGGGT TATCTCGAC- --CTATACAT 317
Sequence logo
360 380 400 420
BI1 TGGCACTGCT CGAGCTGTTT TCCTTAAAGA TGTTCCTAGT GTTGCAATTA GTAAACTTTT CCCAGGGG-- 418
BI2 TGGCACTGCT CGAGCTGTTT TCTTTAAAGA TGTTCCTAGT GTTGCAATTA GTAAACTTTT CCCAGGGG-- 418
BI4 TAACCAAGAT CGAGCCGTTT TCCTTAAAGA TGTTCCTAGT GTTGCAACCC GTGATCTTTT CAAAGGGGTG 417
BI5 TGGCAATGAT CGAGCCGTTT TCCTTAAAGA TGTTCCTAGT GTTGCAACCC GTGATCTTTT CAAAGGGGTG 417
BI6 TAACCAAGAT CGAGCCGTTT TCCTTAAAGA TGTTCCTAGT GTTGCAACCC GTGATCTTTT CAAAGGGGTG 417
BI3 GGACAAAGAT CGAGCCGTTT TCCTTGCCGA GGCTCCTAGT GTTGCAACCC GTCATCTTTT CCCAGGGGTG 417
BI7 GGACAAAGAT CGAGCCGTTT TCCTTGCCGA GGTTCCTAGT GTTGCAACCC GTCATCTTTT CCCAGGGGTG 387
Sequence logo
440 460 480
BI1 -CTATACGTG AAAATTTATC ATTTGATGGC AGCTATACAA AACTTCAAAC TAATGCCGGT GTGACTAGAG 487
BI2 -CTATACGTG AAAATTTATC ATTTGATGGC AGCTATACAA AACTTCAAAC TAATGCCGGT GTGGATAGAG 487
BI4 ACTAATCGTG AACCGTTAAA ATTTGATGGA ACTTACGGAA ACCTTCAAAA GAATGCTGCT GTGACTAGAG 487
BI5 ACTGATCGTG TACCGTTAAA ATTTGATGGA ACTTACGGAA ACCTTCAAAA GAATGCTGGT GTGACTAGAG 487
BI6 ACTAATCGTG AACCGTTAAA ATCTGATGGA ACTTACGGAA ACCTTCAAAA GAATGCTGCT GTGACTAGAG 487
BI3 ACAAATCGTG AAATGTTAAC ATTTTCTGGA CACTATAAAA CCCTTCAAGA GGCTGCCAAA GTGAATAGAG 487
BI7 ACAAATCGTG AAATGTTAAC ATTTTCTGGA CACTATAAAA CCCTTCAAGA GGCTGCCAAA GTGAATAGAG 457
Sequence logo
500 520 540 560
BI1 AGAATCTCGA TCTAGGGGTT AACAATTTGG GATTTGCCAT TGAAGCAATC TATGGTAAAG CGCTAAGTAA 557
BI2 AGAATCTCGA TCTAGGGGTT AACAATTTGG GATTCGCCAT TGAAGCAATC TATGGTAAAT CGTTAAGTAA 557
BI4 AGAATCTCGA TCTAGGGGTT AACAATTTGG GATTTGCCAT TGAAGCAATC TATGGTAAAG CAATAAATAA 557
BI5 AGAATCTCGA TCTAGGGGTT AACAATTTGG GATTTGCCAT TGAAGCAATC TATGGTAAAG CGATAAGTAA 557
BI6 AGAATCTCGA TCTAGGGGTT AACAATTTGG GATTTGCCAT TGAAGCAATC TATGGTAAAG CAATAAATAA 557
BI3 AGAATCTCGA ACTGGGGGTT AACAAATTGG GATTTGCCAT TGAATCAATC GATGGTAAAG CGTTAAATGG 557
BI7 AGAATCTCGA ACTGGGGGTT AACAAATTGG GATTTGCCAT TGAATCAATC TATGGTAAAG CGTTAAATGG 527
Sequence logo

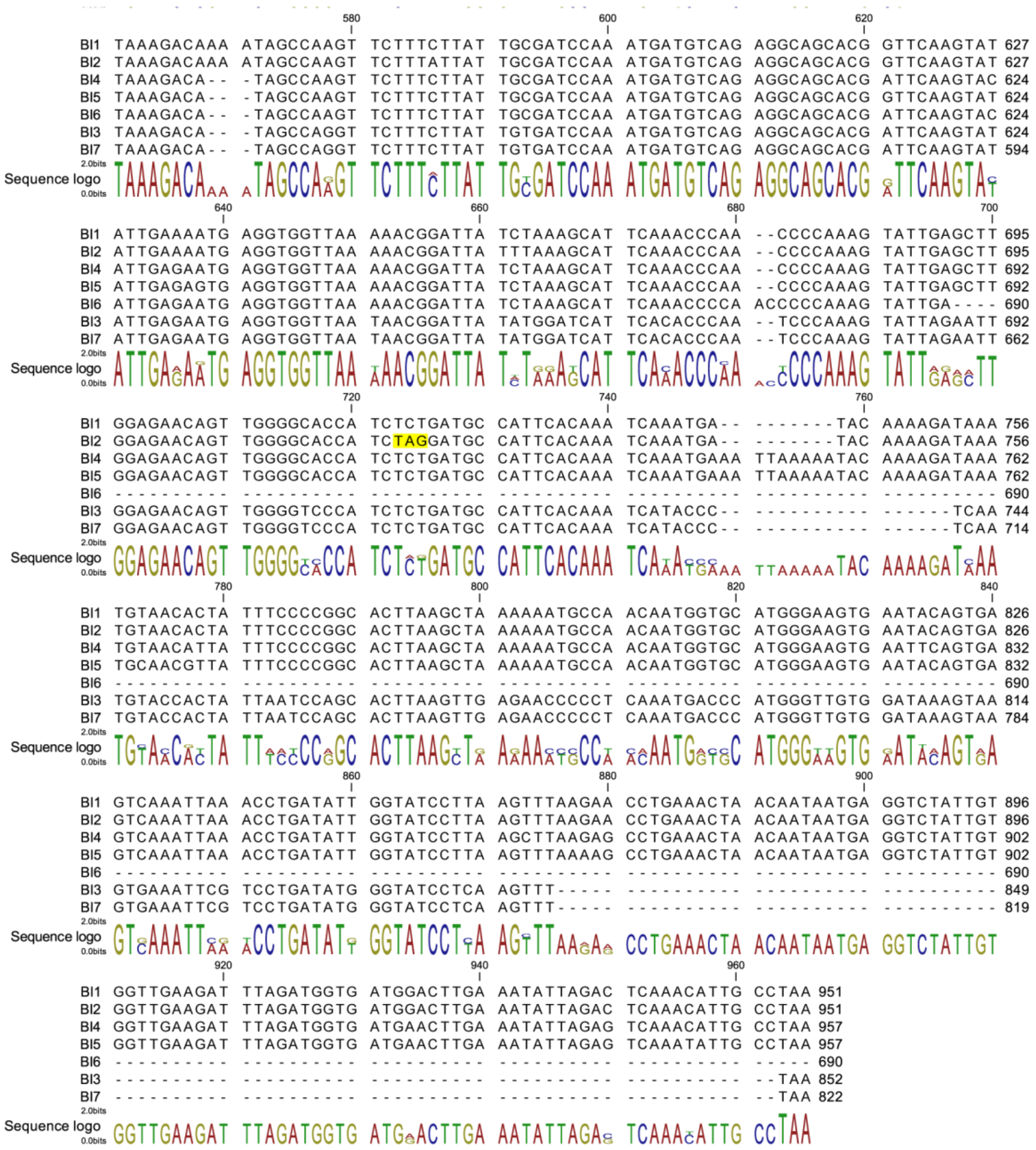

Figure S1. The 3′ end nucleotide sequence alignment of B. glabra isoforms. The yellow shade in BI2 means the stop codon, leading to the shorter amino acid sequence of BI2.

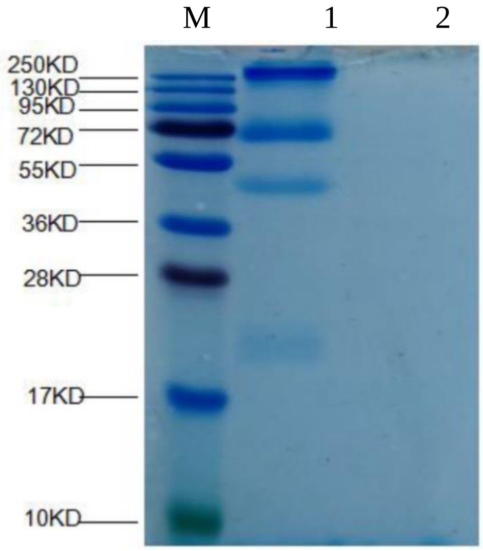

Figure S2. Antibody coupling effect detection. M: protein marker, 1: anti-Bou, 2: coupled flow through.

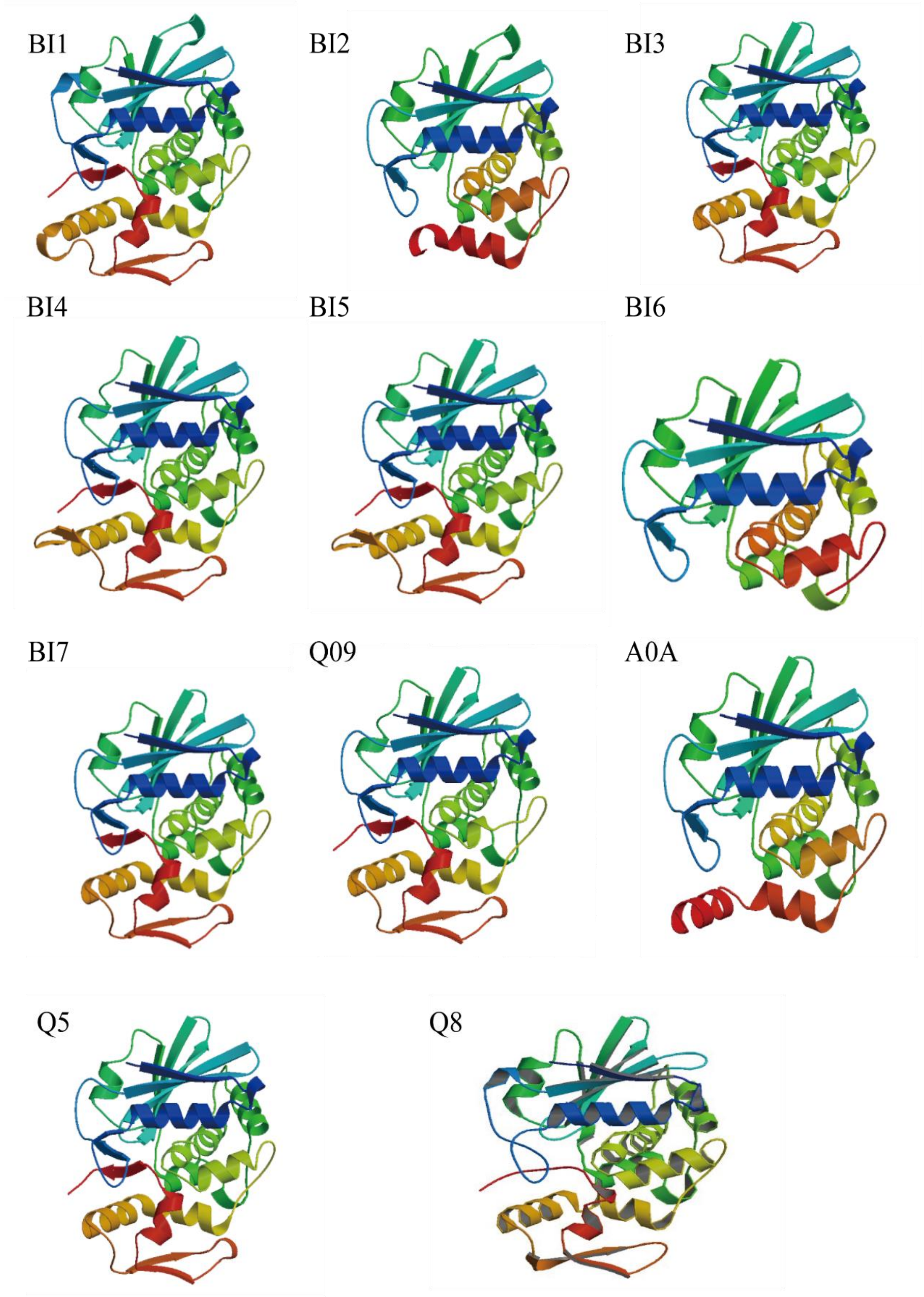

Figure S3. The three-dimensional structure of Bougainvillea RIPs by Swiss-model homologous modeling. Cyan means the N-terminal area and reddish yellow indicates the C-terminal area.
